# One of the Nine Doublet Microtubules of Eukaryotic Flagella Exhibits Unique and Partially Conserved Structures

**DOI:** 10.1371/journal.pone.0046494

**Published:** 2012-10-10

**Authors:** Jianfeng Lin, Thomas Heuser, Kangkang Song, Xiaofeng Fu, Daniela Nicastro

**Affiliations:** Biology Department, Rosenstiel Center, Brandeis University, Waltham, Massachusetts, United States of America; Stanford University School of Medicine, United States of America

## Abstract

The axonemal core of motile cilia and flagella consists of nine doublet microtubules surrounding two central single microtubules. Attached to the doublets are thousands of dynein motors that produce sliding between neighboring doublets, which in turn causes flagellar bending. Although many structural features of the axoneme have been described, structures that are unique to specific doublets remain largely uncharacterized. These doublet-specific structures introduce asymmetry into the axoneme and are likely important for the spatial control of local microtubule sliding. Here, we used cryo-electron tomography and doublet-specific averaging to determine the 3D structures of individual doublets in the flagella of two evolutionarily distant organisms, the protist *Chlamydomonas* and the sea urchin *Strongylocentrotus*. We demonstrate that, in both organisms, one of the nine doublets exhibits unique structural features. Some of these features are highly conserved, such as the inter-doublet link i-SUB5-6, which connects this doublet to its neighbor with a periodicity of 96 nm. We also show that the previously described inter-doublet links attached to this doublet, the o-SUB5-6 in *Strongylocentrotus* and the proximal 1–2 bridge in *Chlamydomonas*, are likely not homologous features. The presence of inter-doublet links and reduction of dynein arms indicate that inter-doublet sliding of this unique doublet against its neighbor is limited, providing a rigid plane perpendicular to the flagellar bending plane. These doublet-specific features and the non-sliding nature of these connected doublets suggest a structural basis for the asymmetric distribution of dynein activity and inter-doublet sliding, resulting in quasi-planar waveforms typical of 9+2 cilia and flagella.

## Introduction

Motile cilia and flagella are important organelles that propel cells or generate fluid flow across tissues, e.g., for mucus clearance in airways. Defects in the assembly or function of these organelles have been linked to a number of human diseases, called ciliopathies, such as primary ciliary dyskinesia and male infertility [1], [2]. Motile flagella are also essential for successful host infection by pathogenic organisms such as *Trypanosoma brucei*, the cause of sleeping sickness [3], [4], making their flagella potential drug targets.

**Figure 1:**
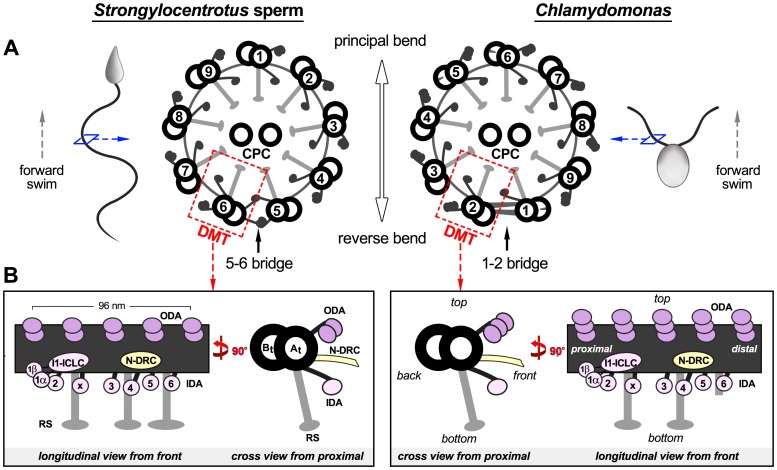
Schematic models of the axoneme organization in the sea urchin *Strongylocentrotus* and the green alga *Chlamydomonas.* (**A**) Two numbering systems for designating the DMTs in 9+2 cilia and flagella are currently in use (left [7], right [15]). The flagella from both organisms have a highly conserved cylindrical arrangement of nine DMTs (red boxes). Each DMT is built from many copies of a 96-nm long unit that repeats along the DMT length. The axonemes are shown in cross-sectional views from the flagellar base (proximal) towards the tip (distal). The locations of the previously described 5–6 bridge (left) and proximal 1–2 bridge (right) are indicated. (**B**) For both organisms, schematic representations of a 96-nm repeat are shown in longitudinal and cross-sectional views; orientations of the 96-nm repeat are maintained in all following figures unless stated otherwise. Other labels: A-tubule (*A_t_*), B-tubule (*B_t_*), inner dynein arms (*IDA 1α*, *1β*, *2–6* and *x*; rose) [43], intermediate chain/light chain complex (*ICLC*), nexin-dynein regulatory complex (*N-DRC*, yellow) [37], outer dynein arm (*ODA*, purple), radial spoke (*RS*, gray) [13], [30].

Most motile cilia and flagella share a highly conserved 9+2 arrangement of microtubules in the axonemal core structure [5], [6]; here, nine doublet microtubules (DMTs) surround two central singlet microtubules of the central pair complex (CPC) and attach to the CPC through radial spokes (Figure 1). In general, two distinct numbering systems are used for designating DMTs of 9+2 cilia and flagella. Historically, numbering was based on the relative position of each DMT with regard to the plane of the CPC [7], [8], which has been adopted for the cilia and flagella of many animals, including sea urchin and mammalian spermatozoa, which have a fixed CPC [7]–[13]. A later numbering system, based on the direction of beating, was proposed for *Chlamydomonas,* given its rotating CPC [14]–[16]. Dynein motor proteins are arranged in two rows, the outer and inner dynein arms (ODA and IDA), along the length of the DMT A-tubule. Driven by ATP hydrolysis, dyneins transmit forces along the B-tubule of the neighboring DMT, causing sliding between adjacent DMTs [17], [18]. Inter-doublet links connecting neighboring DMTs, such as the nexin-dynein regulatory complex (N-DRC), are thought to restrict this sliding displacement between DMTs and thus convert the inter-doublet sliding into a bending motion of the axoneme.

The generation of typical beating patterns for cilia and flagella, such as the quasi-planar motility of many 9+2 cilia and flagella, requires spatial control of dynein-driven inter-doublet sliding around the axoneme [19]–[21]. In vitro DMT sliding studies using axonemes from *Chlamydomonas,* a unicellular green alga, and from sea urchin sperm have indicated that inter-doublet sliding occurs predominantly between specific subsets of DMTs [12], [14], [22]. Significant progress has been made in dissecting axonemal complexes that regulate dynein activity in general, such as the CPC [23]–[27], the radial spokes [13], [27]–[30], the I1 inner dynein [31]–[34], the N-DRC [35]–[38], and the calmodulin and spoke associated complex (CSC) [39], [40]. However, the mechanism for inducing sliding only between specific subsets of DMTs is not well understood. Previous reports have suggested that the CPC distributes asymmetric regulation signals to define localized DMT sliding through radial spokes [26]. However, the CPC/radial spoke system cannot be the only source of asymmetric signaling, because ATP-induced DMT sliding results in the selective extrusion of specific DMTs even in radial spoke-defective *Chlamydomonas* mutants [14], [26]. This suggests that, besides radial spoke signaling, local differences in the composition and/or arrangement of axonemal structures also contribute to the selective activity of dynein and the sliding between DMT subsets.

Previous classical electron microscopy (EM) studies have observed some doublet-specific structures in motile cilia and flagella. Most notable are two inter-doublet bridges; the first doublet-specific bridge discovered was the two-part 5–6 bridge that links DMTs 5 and 6 (using the numbering system that is based on the CPC position) in the axonemes of many animal cilia and flagella, e.g., sea urchin sperm flagella [7], [12] and mollusk paddle and gill cilia [9], [10], [41] (Figure 1A). However, the 3D structure of the 5–6 bridge remains unknown. A second doublet-specific bridge was described as a similar two-part bridge in the proximal quarter of *Chlamydomonas* flagella, but was named the 1–2 bridge, because it connects DMTs 1 and 2 (using the numbering system that is based on the direction of the principal bend) (Figure 1A). The evolutionary relationship between these inter-doublet bridges remains unknown. Another well-known doublet-specific structure is the beak (B-tubule projection) that is only observed in the B-tubules of DMTs 1, 5 and 6 in the proximal half of *Chlamydomonas* flagella [15], [42].

Recent cryo-electron tomography (cryo-ET) studies have described two new doublet-specific structures and have provided remarkable new details of some of the above-mentioned doublet-specific features; these include dynein arm IAX [16], [43] and the radial spoke joist (RSJ) in sea urchin flagella [13], as well as new details regarding the proximal 1–2 bridge [16] and the beak [16], [43]–[45]. Still lacking is an understanding of how these structural differences contribute to the coordination of dynein activity on different DMTs, the associated formation of axonemal bending, different bending patterns, or changes in waveforms in response to environmental stimuli.

In this study, we used cryo-ET and image processing techniques that preserve doublet-specific differences in subtomogram averages, to individually analyze each of the nine DMTs from the flagella of two evolutionarily distant organisms, *Chlamydomonas* and *Strongylocentrotus*. The flagella of both organisms exhibit quasi-planar bending direction under normal conditions; however, *Chlamydomonas* exhibits an asymmetric “breast stroke” often referred to as a cilia-like waveform, whereas the sea urchin sperm flagellum generates a sinusoidal waveform. Our results provide the 3D structures of all nine DMTs from both organisms, revealing that *Chlamydomonas* DMT1 and *Strongylocentrotus* DMT5 exhibit unique structural features that differ from those of the other DMTs. Although some of these features appear to be non-homologous, others are conserved, suggesting a unifying numbering system for indexing DMTs in eukaryotic cilia and flagella. Our observations provide new insights into the role of doublet-specific structures for coordinating the asymmetric distribution of DMT sliding during bend formation.

## Materials and Methods

### Specimen Preparation

Live sea urchins (*Strongylocentrotus purpuratus*) were purchased from Monterey Abalone Co. (Monterey, CA). Spawning was induced by injecting 1 to 2 ml of 0.5 M KCl into the perivisceral cavity [46], and sperm were collected and kept on ice without dilution in artificial seawater to prevent sperm activation [47].

*Chlamydomonas* axonemes were isolated from several *C. reinhardtii* strains (Table 1) as previously described [30]. Briefly, cells growing in liquid Tris-acetate-phosphate medium [48] were harvested, washed, and resuspended in a minimal medium containing 10 mM 4-(2-hydroxyethyl)-1-piperazineethanesulfonic acid (HEPES), pH 7.4, 1 mM SrCl_2_, 4% sucrose, and 1 mM dithiothreitol. The flagella were detached from the cells using the pH-shock method [42]. After adding 5 mM MgSO_4_, 1 mM ethylene glycol tetraacetic acid (EGTA), 0.1 mM EDTA, and 100 µg/ml aprotinin, pepstatin, and leupeptin to the solution, the flagella were separated from the cell bodies by centrifuging for 10 min at 1,800 × g and 4°C and purified by two additional centrifugation steps at 2,400 × g for 10 min at 4°C with a 20% sucrose cushion. Purified flagella were demembranated with 0.1% Nonidet P-40 (Sigma-Aldrich, St. Louis, MO) or 1% IGEPAL CA-630 (Sigma-Aldrich). The axonemes were collected by centrifugation at 35,000 × g for 1 h at 4°C, and were resuspended in 10 mM HEPES, pH 7.4, 25 mM NaCl, 4 mM MgSO_4_, 1 mM EGTA, and 0.1 mM EDTA.

**Table 1:** *Chlamydomonas* strains used in this study and presence of discussed features.

			Number of Tomograms			
Strain	Swimming	Reference	analyzed	i-SUB5-6	proximal 1–2 bridge	
	phenotype			present	present	absent
**WT** (CC-125, 137c)	WT waveform, fast swimming	51, 77	19	19	5	14
**pWT** (*pf2-4::PF2-GFP* a, *ida6::IDA6-GFP* b, *ida6::IDA6-SNAP* c)	WT waveform, fast swimming	34, 37	11	11	3	8
**I1 mutant** *(pf9-3, CC-3913)*	Slow swimming	51, 55	7	7	1	6
**N-DRC mutants** (*sup-pf-3,* *CC-1399, pf2*)	Abnormal waveform, slow swimming	36, 37	8	8	2	6
**N-DRC mutant also lacking IA4**(*ida6, CC-3090*)	Abnormal waveform, slow swimming	78	6	6	2	4

Some of the tomograms from these strains were previously used with different image processing methods to analyze other axonemal complexes [30], [37], [51].

a Strain provided by Raqual Bower and Mary E. Porter (University of Minnesota). The N-DRC defective *pf2* mutant strain [36] was rescued with a GFP-tagged WT *PF2* gene. The resultant strain, *pf2-4::PF2-GFP,* is structurally and phenotypically indistinguishable from WT.

b Strain provided by Douglas Tritschler and Mary E. Porter (University of Minnesota). The N-DRC defective *ida6* mutant strain [78] was rescued with a GFP-tagged WT *IDA6* gene and the resulting *ida6::IDA6-GFP* strain has the same motility and structure as WT.

c Strain provided by Douglas Tritschler and Mary E. Porter (University of Minnesota). The N-DRC defective *ida6* mutant strain [78] was rescued with a SNAP-tagged WT *IDA6* gene. The resultant strain, *ida6::IDA6-SNAP*, is structurally and phenotypically indistinguishable from WT.

Quantifoil holey carbon grids (Quantifoil Micro Tools GmbH, Jena, Germany) were glow discharged at −40 mA for 30 sec and then coated with 10-nm colloidal gold (Sigma-Aldrich, St. Louis, MO). Intact and actively beating *Strongylocentrotus* sperm cells (i.e. ATP inherently present in the flagella) or quiescent *Chlamydomonas* axonemes (no ATP present in the buffer) were applied to the grid together with a tenfold-concentrated 10-nm colloidal gold solution. After blotting from the front side with Whatman #1 filter paper (Whatman, Piscataway, NJ) for ∼2 s, the grid was immediately plunge-frozen in liquid ethane using a home-made plunge-freezing device. The vitrified sample was then stored in liquid nitrogen until examined by EM.

### Cryo-ET

Single axis tilt-series of images were taken using a Tecnai F30 transmission electron microscope (Tecnai F30; FEI, Hillsboro, OR) equipped with a post-column energy filter (Gatan, Pleasanton, CA). Cryo samples were transferred to the microscope using a cryo holder (Gatan) and imaged at 300 keV, at −6 or −8 µm defocus, under low dose conditions, and in the zero-loss mode of the energy filter (20 eV slit width). Tilt-series of images were acquired using the SerialEM image acquisition software [49] and collected from approximately −65 to +65° in steps of 1.5–2.5°. The cumulative electron dose per tilt series was limited to ∼100 e/Å^2^. All images were recorded on a 2 k×2 k charge-coupled device camera (Gatan) at a nominal magnification of 13,500, resulting in a pixel size of ∼1 nm.

### Image Processing

**Figure 2:**
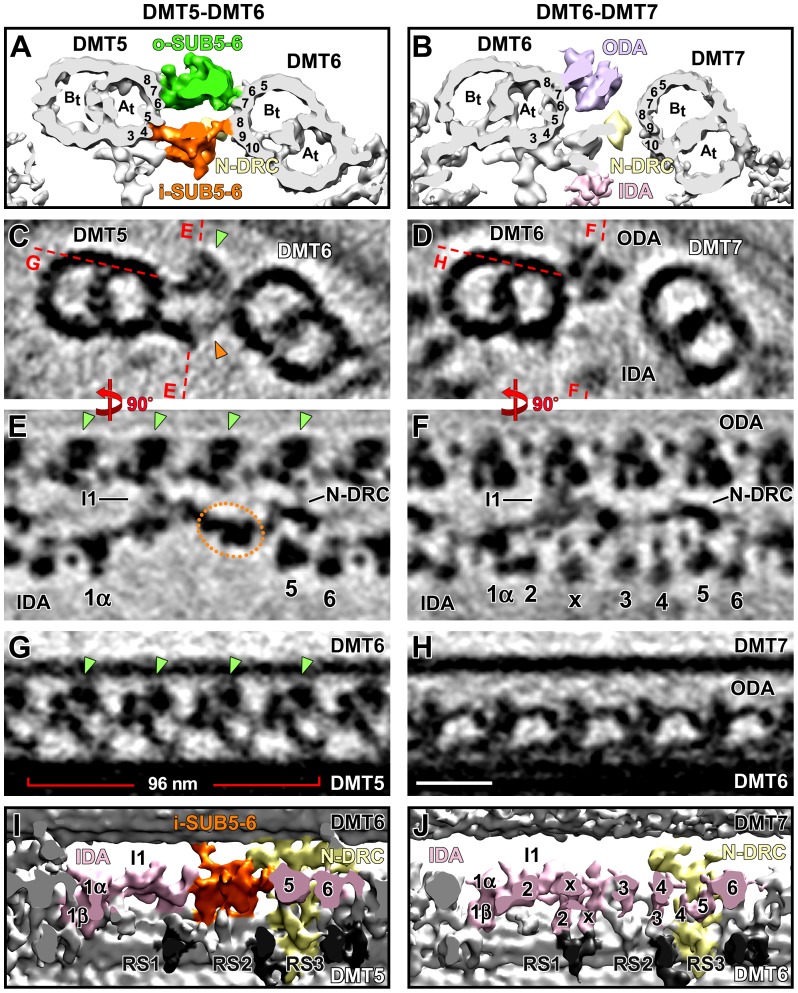
Distinct structural features in the distal 7/8^th^ of *Strongylocentrotus* DMT5 revealed by cryo-ET. Isosurface renderings (A, B, I, and J) and tomographic slices (C–H) show unique structural features present only on DMT5 (left column) in cross-sectional (A–D) and longitudinal views from the front (E and F) and the bottom (G–J). The other eight DMTs are similar to each other but distinct from DMT5. For comparison, the averages of DMT6 are presented in the right column (see also Figure S1 and Movie S1). (**A–D**) Cross-sectional views of SUB5-6, which is present only on DMT5. SUB5-6 consists of two parts: the outer o-SUB5-6 (green) and the inner i-SUB5-6 (orange). The red dashed lines in (C and D) indicate the locations of the tomographic slices shown in (E–H). Note that the center of axonemal repeats shifts longitudinally between neighboring doublets; therefore the longitudinal positions of the slices shown in (A and C) and (B and D) are not identical. (**E** and **F**) Several IDAs (IA2, 3, 4, IAX) are missing from DMT5, and the ODAs are substituted by o-SUB5-6. The dotted circle in (E) outlines the i-SUB5-6 complex that is missing from other DMTs, as shown for DMT6 (F). (**G–J**) Details of the outer and inner SUB5-6 structures. The o-SUB5-6 complexes (green arrowheads) are morphologically distinct from the ODAs, but share the same 24-nm periodicity (compare G and H); i-SUB5-6 (orange) has a 96-nm periodicity and makes clear connections with I1 dynein and N-DRC while linking DMT5 to DMT6. Other labels: A-tubule (*A_t_*), B-tubule (*B_t_*), inner dynein arms (*IDA 1α*, *1β*, *2–6, x*; rose), nexin-dynein regulatory complex (*N-DRC*, yellow), outer dynein arm (*ODA*, purple), radial spoke (*RS*, dark-gray). Cross-sections are viewed from the proximal, and in longitudinal views proximal is on the left. The sea urchin DMT numbers are according to [7]. The protofilaments are numbered after [53]. Scale bar (H): 25 nm.

**Figure 3:**
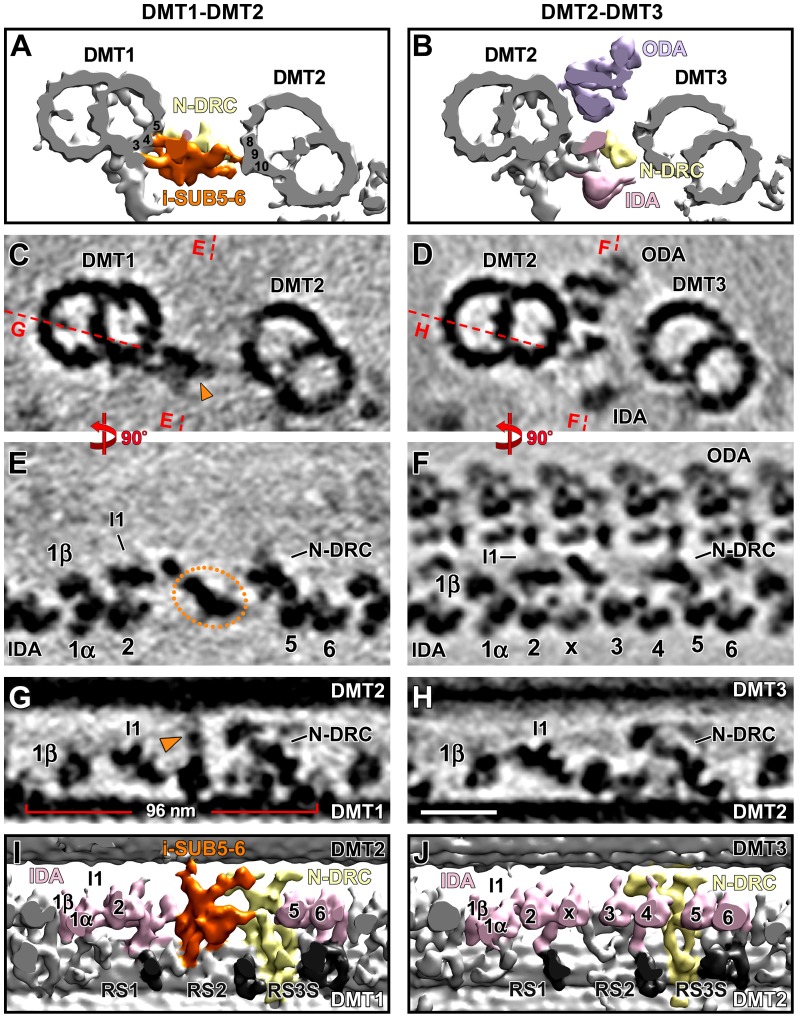
Distinct structural features in the distal three quarters of *Chlamydomonas* DMT1. Isosurface renderings (A, B, I, and J) and tomographic slices (C–H) show i-SUB5-6 and the missing IDAs on *Chlamydomonas* DMT1 (left column) in cross-sectional (A–D) and longitudinal views from the front (E and F) and the bottom (G–J). The structure of DMT1 is unique and different from DMTs 2–9, which have a similar structure; DMT2 is shown for comparison in the right column (see also Figure S1 and Movie S3). (**A–D**) Cross-sectional views of the i-SUB5-6 complex (orange), which is present on DMT1 only and links DMT1 to DMT2. The ODAs are missing from DMT1 but are present on all other DMTs. The red dashed lines in (C and D) indicate the locations of the tomographic slices shown in (E–H). Note that the beak structure in the B-tubule of DMT1 in (A and C) has previously been reported to be present in the proximal half of the flagellum [15], and is therefore still visible in this average of the distal three quarters of the axoneme. (**E** and **F**) Several IDAs (IA3, 4, and IAX) are absent on DMT1 (E) but present on DMT2 (F). The orange dotted circle in (E) outlines the i-SUB5-6 complex that is present on DMT1, but absent from DMT2 (F). (**G–J**) Details of the i-SUB5-6 structure (orange) demonstrate that it has a periodicity of 96 nm and makes clear connections with both I1 dynein and N-DRC while linking DMT1 to DMT2. Other labels: A-tubule (*A_t_*), B-tubule (*B_t_*), inner dynein arms (*IDA 1α*, *1β*, *2–6* and *x*; rose), nexin-dynein regulatory complex (*N-DRC*, yellow), outer dynein arm (*ODA*, purple), radial spoke (*RS*, dark-gray). The *Chlamydomonas* DMT numbers are according to Hoops and Witman [15]. The protofilaments are numbered after Linck and Stephens [53]. Scale bar (H): 25 nm.

**Figure 4:**
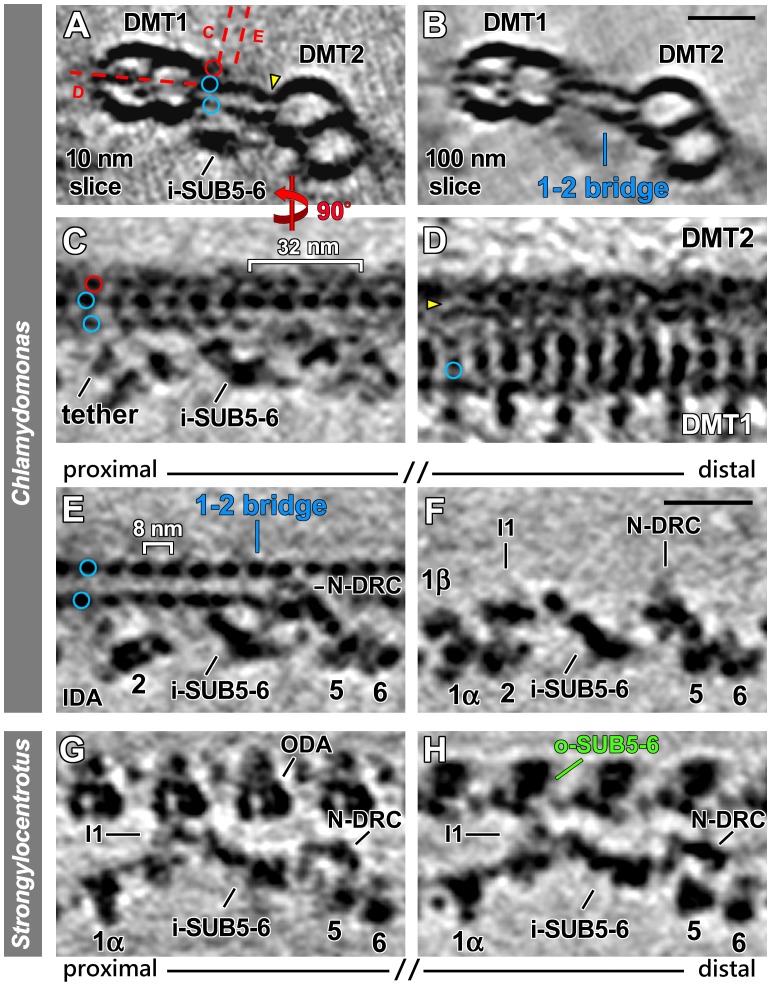
Distinct structural features in specific regions of *Chlamydomonas* DMT1 and *Strongylocentrotus* DMT5. (**A–E**) The 1–2 bridge in the proximal quarter of *Chlamydomonas* DMT1. Tomographic slices show the proximal 1–2 bridge between DMTs 1 and 2 in *Chlamydomonas* pWT in cross-sectional (A and B) and longitudinal views from the front (C and E) and the bottom (D). Red dashed lines in (A) indicate the locations of tomographic slices shown in (C–E). The 1–2 bridge consists of two parts that link DMTs 1 and 2; each part consists of a row of periodic densities (blue circles in A, and C–E). The connection of the top row to DMT2 is highlighted (yellow arrowheads in A and D). The red circles in (A and C) indicate an additional row of periodic but shorter densities that do not reach DMT2. Note that the I1 dynein is absent from the proximal quarter of DMT1 and only a small neighboring density, the I1 *tether* [34], is visible (C and E). (**F**) A tomographic slice from the distal part of DMT1 is shown for direct comparison with the proximal slice in (E). Note that the distal region of *Chlamydomonas* DMT1 lacks the 1–2 bridge, while the I1 dynein is present. (**G** and **H**) Tomographic slices in front view show a comparison between the proximal 1/8^th^ (G) and distal 7/8^th^ (H) of the *Strongylocentrotus* DMT5. While in the very proximal region regular ODAs are present, they are substituted by o-SUB5-6 structures in the distal 7/8^th^ of the flagellum. Note that along the entire length of DMT5 i-SUB5-6 is consistently present, while several IDAs (IA 2–4, IAX) are lacking. Scale bars are 25 nm (scale bar in B valid for A and B; scale bar in F valid for C–H).

**Figure 5:**
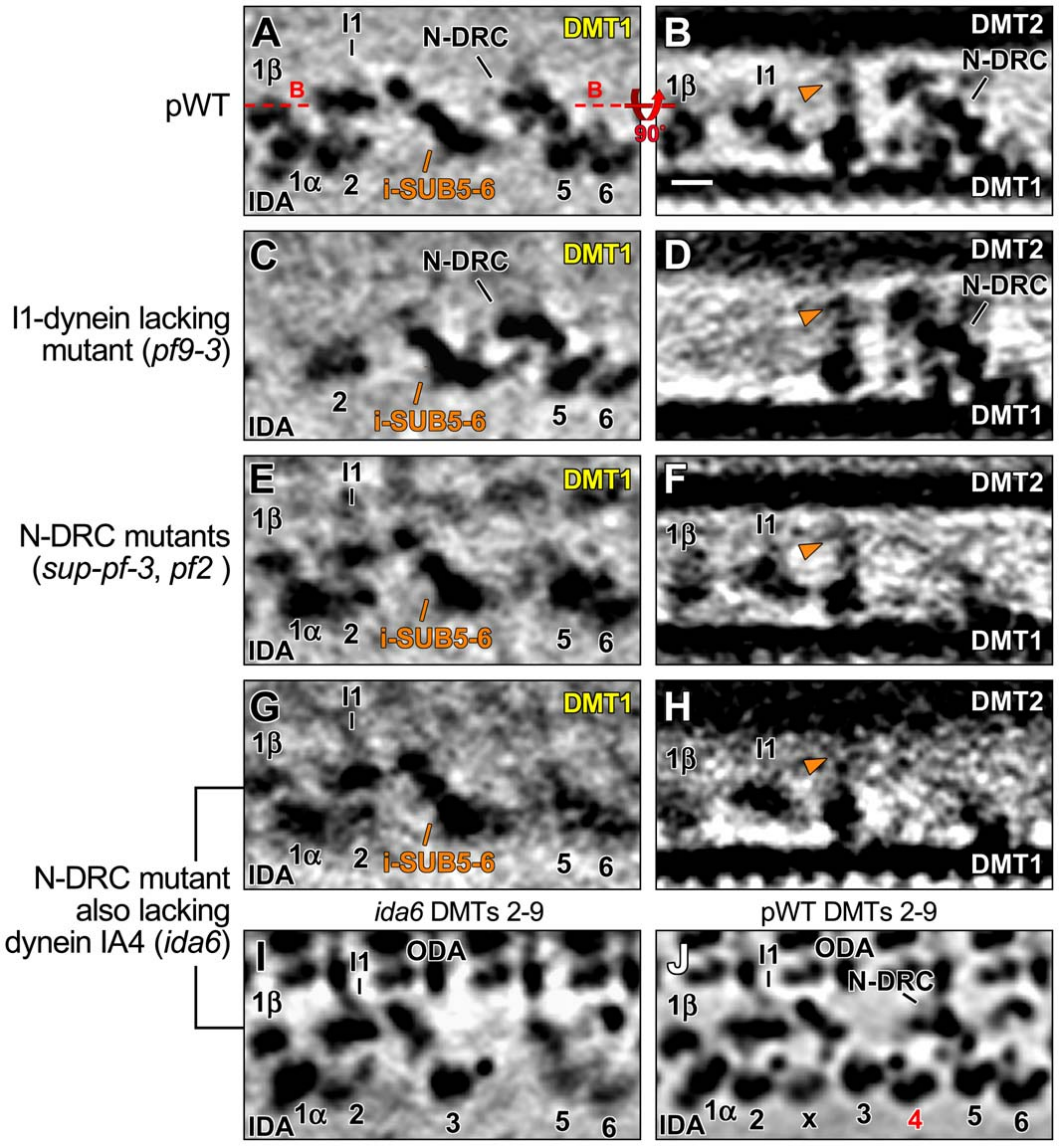
i-SUB5-6 is present in *Chlamydomonas* I1 mutants and N-DRC mutants. (**A–H**) A comparison of tomographic slices from averages of the distal part of DMT1 from *Chlamydomonas* pWT (A and B), I1-dynein-lacking mutant *pf9-3* (C and D), a combined average of the N-DRC mutants *sup-pf-3* and *pf2* (E and F), and the dynein-IA4-lacking mutant *ida6* (G–I) indicates that the lack of I1 dynein or IA4 (dynein e) or a dramatic reduction in the N-DRC has little effect on the i-SUB5-6 structure (orange arrowheads). The tomographic slices were viewed from the front (left column) and the bottom (right column) and show the same locations of the axonemal repeat as shown in Figure 3E and 3G, respectively. The orientation of the tomographic slice shown in (B) is indicated by red dashed lines in (A). (**I** and **J**) Tomographic slices from combined averages of DMT2-9 show the absence of IA4 in the *ida6* axoneme (I) compared to pWT (J). Scale bar (B): 10 nm.

3D tomograms were calculated using the IMOD software package [50] with gold fiducial marker alignment and weighted back projection. Only tomograms of intact and non- or mildly compressed flagella and axonemes were used for further image processing. Some of the tomograms were previously utilized for the analysis of other axonemal complexes [13], [30], [37], [51] but with different image processing methods. We used several well-established criteria for determining the polarity of the axonemes in the tomograms, such as the sequence of axonemal structures along the length of the A-tubule (I1 dynein proximal of the N-DRC), and the clockwise sequence of A-tubules connecting to neighboring B-tubules when the flagellum is viewed in cross-section from the proximal end. Doublet-specific averaging was carried out using PEET (Particle Estimation for Electron Tomography) software [51]. Subtomograms containing the 96-nm axonemal repeats were extracted from tomograms (summarized in Table 1), aligned, and separately averaged in 3D for each DMT. The DMT numbers were identified based on unique structures such as the 5–6 bridge in sea urchin [7] or the 1–2 bridge and missing ODAs in *Chlamydomonas* [15], [16]. The DMT region in which each tomogram was recorded could be determined either directly using the relative location to the sperm head of the sea urchin sperm flagellum, or indirectly by well-established structural markers of particular regions of the *Chlamydomonas* flagellum [15]. All axonemal repeats of the same DMT that share the same structural features were combined and averaged from all tomograms of the same strain. The IMOD software package [50] and the UCSF Chimera package [52] were used for visualization of the tomographic slices and 3D visualization by isosurface rendering, respectively (Figures 2, 3, 4, 5; Figures S1, S2, S3; Movies S1, S2, S3).

## Results

### DMT5 is Distinct from the Other DMTs in the *Strongylocentrotus* axoneme

DMT sliding is the driving mechanism of ciliary and flagellar motility. However, to generate bending, this sliding must occur in a well-orchestrated fashion between selected DMT pairs. To explore the structural differences that could be involved in defining which DMTs slide and/or which do not, we performed cryo-ET and doublet-specific subtomogram averaging of *Strongylocentrotus purpuratus* sperm flagella. We separately examined thirteen cryo-tomographic reconstructions of sea urchin flagella and individually averaged the 96-nm axonemal repeats from each DMT. We observed several distinct features that allowed us to identify and combine corresponding DMT from different flagella. The combined doublet-specific averages of each DMT provide unprecedented 3D structural detail and reveal the unique structural features of DMT5 that differ from those of the other DMTs (Figure 2, compare DMTs 5/6 in the left column to DMTs 6/7 (for example) in the right column; Figure S1; Movie S1). Since the sea urchin sperm cells were frozen intact with heads, we were able to determine the location of every tomogram based on its relative distance to the sperm head. Therefore, we also calculated doublet-specific averages for specific flagellar regions. We found that the most proximal 1/8^th^ of DMT5 (5 tomograms) lacks one of the differences that distinguishes the remaining 7/8^th^ of DMT5 (8 tomograms) from all other doublets.

Attached to the A-tubule of DMT5 are structures previously observed in cross-sectional EM views, which are collectively called the 5–6 bridge, connecting DMTs 5 and 6 in many flagella [7], [9]–[12], [41], including sea urchin flagella (Figure 2, left column). Our 3D doublet-specific average of DMT5 revealed that the two parts of this sea urchin bridge between DMTs 5 and 6 (SUB5-6) look markedly different. In the cross-sectional view (Figure 2A, C), both parts of SUB5-6 together have a diamond-shaped outline, consistent with previous studies [7], [9]–[12], [41]. The outer part, o-SUB5-6, substitutes the outer dyneins in the distal 7/8^th^ of DMT5 (Figure 4H), while regular ODAs are present in the proximal 1/8^th^ (Figure 4G). o-SUB5-6 extends from protofilaments A6, A7, and A8 (protofilament numbering according to Linck and Stephens [53]) of the A-tubule of DMT5 and attaches to B6 and B7 of the B-tubule of DMT6. In contrast, the inner part, i-SUB5-6, is present along the entire length of DMT5, extending from protofilaments A4 and A5 of DMT5 to B8 and B9 of DMT6 (Figure 2A; Movie S1). Another significant difference between the two SUB5-6 parts is that the i-SUB5-6 structure repeats with a periodicity of 96 nm along the length of the doublet (Figure 2E, I), whereas the o-SUB5-6 structure repeats with a periodicity of 24 nm (Figure 2E, G). Although the periodicity and location of o-SUB5-6 resemble those of ODAs (Figure 2F, H), o-SUB5-6 structures display a unique morphology (compare Figure 2A, C, E, G to Figure 2B, D, F, H; Movie S2), including a large density that is connected to DMT6 rather than the narrow connections of the ODAs to the neighboring DMT through their thin coiled-coil stalks (Figure S2A, B). In addition to linking DMTs 5 and 6, both i-SUB5-6 and o-SUB5-6 connect to the I1 dynein and the N-DRC. In each axonemal repeat, one o-SUB5-6 complex per axonemal repeat connects to the I1 dynein through the proximal outer-inner-dynein (OID) linker, and the most distal o-SUB5-6 complex links to the N-DRC via the distal OID linker. In contrast, only one i-SUB5-6 complex is present per repeat, which connects proximal to the I1 dynein and distal to the N-DRC (Figure 2E, I).

There are two more obvious structural differences present along the entire length of DMT5 in comparison to the other eight DMTs. Typically, the inner row of dynein arms of eukaryotic axonemes contains the double-headed I1 complex (dynein f) and five to six single-headed dyneins (IA2-6 and IAX; dyneins a-e, g) (Figure 2F, J) [13], [16], [43], [45], [51], [54]. Here, we show that in *Strongylocentrotus* axonemes, DMT5 lacks 4 of these single-headed dyneins: IA2-4 and IAX (Figures 2E, I, 4G). Among these IA2-4 are only missing from the unique DMT5, while IAX is also missing from DMTs 1 and 9 (Table 2; Figure S1). The N-DRC on DMT5 appears to form a more substantial connection to the neighboring DMT than the N-DRC of the other DMTs (compare Figure 2I and 2J).

**Table 2:** Correlation of DMT numbering systems and doublet-specific features.

		Reference	Doublet number								
DMT numbering system	Unified numbering	this study	D-I	D-II	D-III	D-IV	D-V	D-VI	D-VII	D-VIII	D-IX
	**Sea urchin (** ***S.p.*** **)**	7	1	2	3	4	5	6	7	8	9
	***Chlamydomonas*** ** (** ***C.r.*** **)**	15	6	7	8	9	1	2	3	4	5
**Doublet-specific features**	***C.r.*** ** & ** ***S.p.*** ** i-SUB5-6, ** ***S.p.*** **o-SUB5-6, ** ***C.r.*** **1–2 bridge**	this study; 7, 15	−	−	−	−	−	−	−	−	−
	***S.p.*** ** dynein IA2,3,4**	this study	+	+	+	+	−	+	+	+	+
	***C.r.*** ** dynein IA3,4**	this study; 16	+	+	+	+	−	+	+	+	+
	***S.p.*** ** dynein IAX**	this study;	−	+	+	+	−	+	+	+	−
	***C.r.*** ** dynein IAX**	this study; 16^a^	+	+	+	−	−	+	+	+	−
	***C.r.*** ** beak**	42	+	−	−	−	+	−	−	−	+
	***C.r.*** ** I1 dynein modification**	34^b^	−	−	−	−	−	−	+	+	−
	***S.p.*** ** RS joist**	13	−	−	+	+	−	−	+	+	+
	***S.p.*** ** RS spur**	13	−	+	−	−	−	−	−	−	−

a Bui et al. [16] reported that in *Chlamydomonas* IAX (dynein b/g) is clearly missing from DMT9, and maybe missing from DMT1– consistent with the data presented here; however, the authors did not report that IAX is greatly reduced at DMT5, as shown here.

b Heuser et al. [34]: in *Chlamydomonas* axonemes the intermediate chain and light chain complex (ICLC) of the I1 dynein contains a doublet-specific protrusion only found on DMTs 3 and 4, but not on the other seven DMT.

### Conserved Structural Features Observed for DMT1 of the *Chlamydomonas* axoneme

Although motile cilia and flagella share a highly conserved 9+2 axoneme core structure in general, structural differences have been reported across different organisms [13], [29], [45]. To better understand the common mechanism of 9+2 ciliary and flagellar motility, our study further examined similarities and diversity in axonemal structures among the two evolutionarily distant flagella model organisms *Strongylocentrotus* and *Chlamydomonas*. One advantage of studying *Chlamydomonas* is the large arsenal of available motility mutants, allowing for a correlation of structural features with phenotypes and functions. Here, we analyzed doublet-specific differences in wild-type *Chlamydomonas* (WT), several mutants, and several *drc*-mutant rescue strains with the WT phenotype, named pseudo wild-type (pWT). Details about the strains used and the features examined are summarized in Table 1. The pWT strains have been studied extensively and are biochemically, structurally, and phenotypically indistinguishable from WT [30], [34], [36], [37], [44]. Using the same doublet-specific averaging techniques described for *Strongylocentrotus* above, we calculated subtomographic averages of the 96-nm axonemal repeats of each individual DMT of the axonemes of all investigated *Chlamydomonas* strains (Figures 3, 4, 5). After determining the doublet identity based on doublet-specific features, such as the presence of a beak or missing ODAs, corresponding DMTs were combined from multiple tomograms. Axonemal averages of the WT and pWT strains provided nearly identical 3D structures (for comparison, see Figure S3), but greater detail was revealed by the averages of the pWT strains due to their higher resolution.

Similar to the *Strongylocentrotus* axoneme, the *Chlamydomonas* axoneme also contains one DMT that exhibits unique structural features similar to sea urchin DMT5 but that differentiate it from the remaining eight DMTs. Following the current DMT numbering system for *Chlamydomonas* flagella, this specialized doublet is termed DMT1 (Figures 3, 4; Figure S1; Movie S3). In contrast to sea urchin sperm that were frozen intact, *Chlamydomonas* axonemes were isolated, making the determination of the region in which tomograms were recorded not as easy. Therefore, we utilized previously established structural markers to identify specific regions of the *Chlamydomonas* flagellum, such as the 1–2 bridge in the proximal quarter and the B-tubule beak in the proximal half of the flagellum [15]. Similar to *Strongylocentrotus* and consistent with previous reports, regional doublet-specific average confirmed that *Chlamydomonas* axonemes exhibit structural heterogeneity along the flagellar length, i.e., DMT1 contains structural features only observed in the proximal region of the axoneme (Figures 3, 4). Some but not all of these unique structures appear to be conserved between *Chlamydomonas* DMT1 and *Strongylocentrotus* DMT5.

The i-SUB5-6 structure observed for *Strongylocentrotus* DMT5 is also present along the entire length of the *Chlamydomonas* DMT1 (Figures 3, 4; Table 1). The location of this structure between the I1 dynein and N-DRC, and the overall structure of *Chlamydomonas* i-SUB5-6 is similar to that of *Strongylocentrotus* (compare Figures 2, 3), including the connection between DMTs 1 and 2 (Figure 3A, C, G, I), as well as to the I1 dynein and N-DRC through its proximal and distal regions (Figure 3I; Movie S3). Another similarity to sea urchin flagella is the absence of ODAs and several single-headed IDAs from the entire length of DMT1 in *Chlamydomonas* axonemes. However, rather than missing four IDAs, *Chlamydomonas* DMT1 lacks only three: IA3, 4, and IAX; IA2 remains present (Figures 3E, I, 4E). As previously reported [16], [43] and similar to sea urchin flagella, IAX is also missing from *Chlamydomonas* DMT9 (Table 2; Figure S1); in addition, we found that IAX is greatly reduced from DMT5. A significant difference from *Strongylocentrotus* DMT5 is that *Chlamydomonas* DMT1 does not contain o-SUB5-6 structures in place of regular ODAs. The ODA region of *Chlamydomonas* DMT1 is either completely vacant in most examined tomograms (Figure 3A, C, E; Movie S3) or, in the proximal region of the axoneme [15], the ODA site is occupied by the structures of the proximal 1–2 bridge, which shares no similarities with o-SUB5-6 (Figure 4, see below for details). Another difference from sea urchin is that in the proximal region, where the proximal 1–2 bridge is present, another IDA is lacking: the double-headed I1 dynein complex (Figure 4E).

### The Proximal 1–2 Bridge on DMT1 of the *Chlamydomonas* axoneme

Although *Strongylocentrotus* o-SUB5-6 is not present in *Chlamydomonas* DMT1, we observed another inter-doublet link, the proximal 1–2 bridge, in approximately one quarter of our *Chlamydomonas* tomograms (Table 1). This is consistent with previous findings of classical EM and cryo-ET studies regarding the presence of a 1–2 bridge in the proximal quarter of the *Chlamydomonas* flagellum [15], [16]. We separately calculated doublet-specific averages of DMT1 for eleven tomograms of pWT strains; then, we combined the three DMT1 averages that included the proximal 1–2 bridge into a proximal axonemal average (Figure 4A, B, C, D, E) and combined the remaining eight tomograms into a distal axonemal average that shows no structures in the ODA region (Figures 3A, C, E, 4F). The proximal axonemal average of *Chlamydomonas* DMT1 provides a detailed 3D structure of the proximal 1–2 bridge (Figure 4A, B, C, D, E).

Consistent with previous studies [15], [16], we found that the proximal 1–2 bridge is composed of two straight linker parts (Figure 4A, B, blue circles), shown in cross-sectional views, which make substantial connections to the B-tubule of DMT2 (Figure 4A, D, yellow arrowheads). Both the outer and inner portions of the proximal 1–2 bridge exhibit an 8-nm periodicity of rungs along the flagellar axis, but the inner portion has a 32-nm-long discontinuity in the region of the N-DRC (Figure 4C, E). This high repetitiveness makes the 1–2 bridge clearly visible in 10-nm- and 100-nm-thick cross-sectional slices through the tomographic average (Figure 4A, B). i-SUB5-6 is also present in the proximal quarter of *Chlamydomonas* DMT1; however, due to its lower periodicity (96 nm) compared to the proximal 1–2 bridge, i-SUB5-6 is difficult to distinguish in the 100-nm tomographic slice (Figure 4B). This explains why only the two-part 1–2 bridge has been reported for *Chlamydomonas* axonemes. Above the two linker parts of the proximal 1–2 bridge, we found a third row of densities with the same 8-nm periodicity (red circles in Figure 4A, C). However, these rungs extend for only approximately 10 nm towards the neighboring DMT and are thus much shorter than the densities in the two rows linking DMTs 1 and 2 (Figure 4A, B).

### Comparison with Mutants Shows that Defects in the N-DRC and I1 Dynein Structures have Little Effect on the i-SUB5-6 Structure

As mentioned above, i-SUB5-6 and its connections to two known regulators of dynein activity, I1 dynein and N-DRC, are consistently observed on *Chlamydomonas* DMT1 and *Strongylocentrotus* DMT5. No i-SUB5-6 mutant has been identified thus far; however, several *Chlamydomonas* mutants have been shown to display specific structural defects in I1 dynein or N-DRC; for example, in the *pf9-3* axoneme, the entire I1 dynein is missing [34], [51], [55]. Similarly, in many *drc*-mutant axonemes, the majority of the N-DRC density is missing, particularly that for the nexin linker and the portion that connects to i-SUB5-6 [35], [37]. In addition, some of these *drc*-mutants also lack dynein IA4 (dynein e), which has been hypothesized to be part of the density here identified as i-SUB5-6 [16].

To determine whether the assembly of i-SUB5-6 is dependent on N-DRC, I1 dynein, or dynein IA4, we compared the axonemal averages of the available I1 and N-DRC mutants in regards to possible affects on the i-SUB5-6 structure (Figure 5). Our results indicate that although I1 dynein is completely missing in the *pf9-3* axoneme, i-SUB5-6 is still present throughout the entire length of DMT1 and exhibits no obvious structural defects (Figure 5C, D). Similarly, in N-DRC mutants, the lack of the N-DRC regions that would usually directly link to i-SUB5-6 does not disrupt the assembly of i-SUB5-6 on DMT1 (Figure 5E, F). In addition, *drc*-mutant axonemes that are known to lack dynein IA4 [36], [37], [44] show that an absence of IA4 has little effect on the assembly of i-SUB5-6 (Figure 5G, H).

## Discussion

Most motile cilia and flagella have a constant bending plane and generate quasi-planar waveforms, which result in faster swimming than helical waveforms [56]. Variations from a quasi-planar to a helical waveform sometimes occur under certain conditions, e.g., in response to light [57] or increased viscosity of the surrounding medium [58]. The DMT pairs DMT5-6 in *Strongylocentrotus* and DMT1-2 in *Chlamydomonas* are located at a plane almost perpendicular to the bending plane (Figure 1) and are known to show little or no inter-doublet sliding [12], [14], [22], [59], [60]. In *Chlamydomonas*, DMT1 is a continuation of the basal body triplet 1, which is part of the three triplets contacted by the distal striated fibers that are important for controlling the rotational orientation of the flagella [15], [61], [62]. Using cryo-ET and doublet-specific subtomogram averaging, we investigated the 3D structures of the nine DMTs from *Strongylocentrotus* and *Chlamydomonas* axonemes, revealing that *Strongylocentrotus* DMT5 and *Chlamydomonas* DMT1 display unique structural features, some of which are conserved between the two organisms. Many of these features are consistent with the provision of robust connections between adjacent DMTs and the prevention of inter-doublet sliding. These features suggest that the functional roles of specific doublets during flagellar motility are structurally pre-determined at the level of the DMTs and associated complexes rather than completely relying only on regulatory signals provided by the CPC/radial spoke system.

### Additional Inter-doublet Links Present on one Unique DMT Suggest a Robust Connection Intended to Resist Inter-doublet Sliding

N-DRC is a well-known inter-doublet link connecting each of the 9 DMTs of an axoneme to its neighboring DMT. It is thought to function in the regulation of dynein activity and the conversion of inter-doublet sliding into axonemal bending [35], [37], [63], [64]. In addition to N-DRC, additional links, such as the 5–6 bridge in the axonemes of echinoderms and mollusks or the 1–2 bridge in *Chlamydomonas*, have previously been observed by classical EM [7], [10], [12], [15], [41]. A recent cryo-ET study reported the first 3D structural details of the *Chlamydomonas* proximal 1–2 bridge [16]. However, 3D details of the 5–6 bridge in echinoderms and mollusks have not been available; therefore, the relationship between these two bridge structures has remained unclear. Here, we observed that the sea urchin 5–6 bridge (SUB5-6) and the *Chlamydomonas* 1–2 bridge exhibit markedly different morphologies and periodicities from each other, and therefore, they most likely form two distinct and non-homologous inter-doublet links.

**Figure 6:**
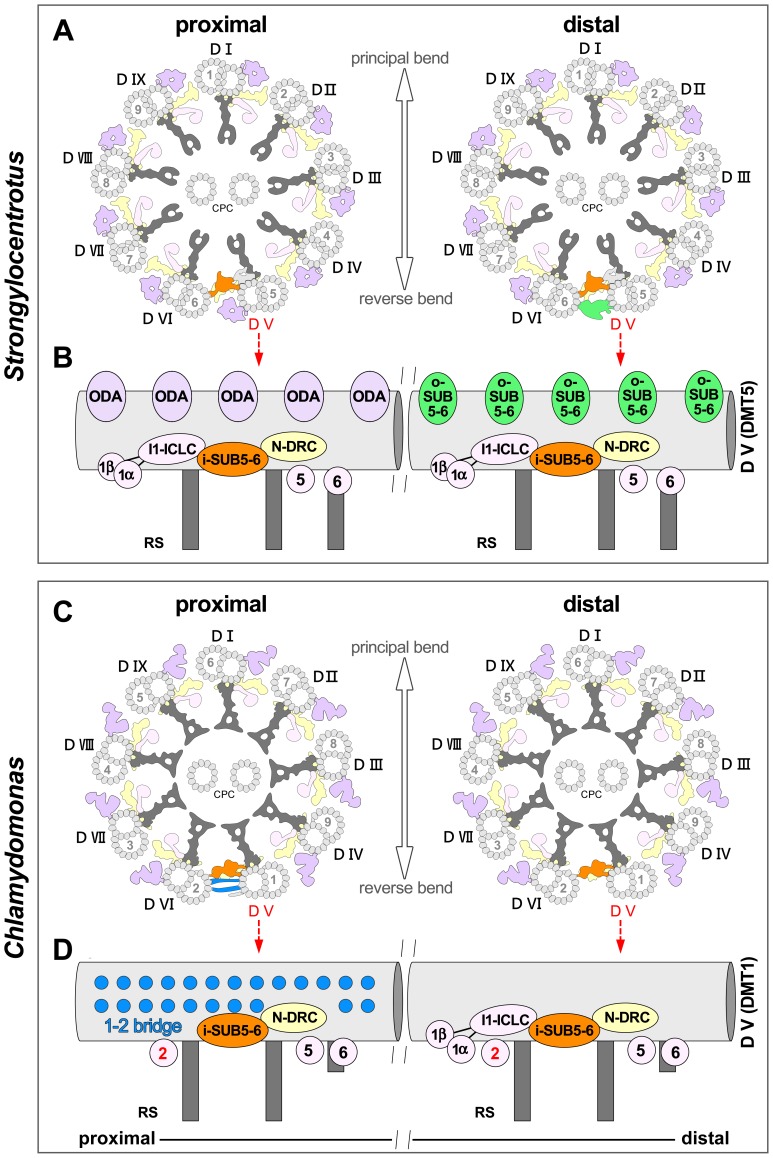
Schematic models of the axonemes and doublets D-V from *Strongylocentrotus* and *Chlamydomonas*. (**A** and **C**) Simplified diagrams of the axonemes viewed in cross-section from proximal correlate the DMTs from *Strongylocentrotus* and *Chlamydomonas.* A unifying numbering convention for the DMTs in eukaryotic 9+2 axonemes is used, based on the observed conserved structural features. This convention facilitates comparisons of DMTs among species and assigns the same doublet number to corresponding DMTs. The previously used numbering systems (Arabic numerals in B-tubules) are also depicted for comparison. Under the new numbering system, the conserved inter-doublet linkers of both species described in this study, are present only on doublet D-V (some of these features connect to D-VI). Double-headed arrows indicate the flagellar beating plane that runs through doublet D-I and between D-V/D-VI that are connected by i-SUB5-6. (**B** and **D**) The diagrams of D-V show longitudinal front views that summarize the conserved structural features. The i-SUB5-6 complex (orange) is present in every 96-nm repeat along the unique DMT D-V and links to the neighboring DMT D-VI. ODAs are absent from the distal 7/8^th^ of *Strongylocentrotus* DMT D-V, and almost from the entire length of *Chlamydomonas* DMT D-V. However, additional inter-doublet links such as the *Strongylocentrotus* o-SUB5-6 complexes (green) or the *Chlamydomonas* proximal 1–2 bridge (blue) are present on DMT D-V, while several IDAs (IA2, 3, 4, and IAX in *Strongylocentrotus*; IA3, 4, and IAX in *Chlamydomonas*) are missing from this DMT.

Intriguingly, we found that the inner part of the 5–6 bridge, i-SUB5-6, is present along the entire length of a specialized DMT in both *Strongylocentrotus* and *Chlamydomonas* flagella (Figures 2, 3, 4, 6), suggesting that i-SUB5-6 is a highly conserved inter-doublet link. The i-SUB5-6 has the same periodicity as N-DRC, and in cross-sectional views, its location is also similar to the position of the N-DRC linker, making it difficult to distinguish i-SUB5-6 from N-DRC using classical EM. This likely explains why the i-SUB5-6 structure has not been described by classical EM studies of *Chlamydomonas* flagella, and the same could be true for many cilia and flagella studies in general. A recent cryo-ET study has also observed the i-SUB5-6 structure (termed IDL3) in *Chlamydomonas* DMT1 [16]. We expect that future cryo-ET studies of 9+2 cilia and flagella of other organisms will also reveal i-SUB5-6 as a conserved feature of one specialized DMT.

Previous classical EM studies did not report any changes of the 5–6 bridge along the length of mussel gill cilia [11]. Our data, however, revealed that the outer part of the 5–6 bridge, o-SUB5-6, is not present in the very proximal region of the flagellum. This is in contrast to the *Chlamydomonas* flagellum, where the proximal 1–2 bridge is limited to only the proximal quarter of the flagellum (Figure 6). Although our data revealed that sea urchin o-SUB5-6 and the *Chlamydomonas* proximal 1–2 bridge are structurally very different and most likely non-homologous, the function of these two massive inter-doublet connections between two adjacent DMTs could still be analogous. The nexin link of N-DRC is thought to either form permanent and elastic connections to the neighboring doublet or connections that translocate when inter-doublet sliding occurs [64]–[66]. All three bridging structures described here, i-SUB5-6, o-SUB5-6 and the 1–2 bridge, could similarly be permanent or transient inter-doublet connections. However, in contrast to the N-DRC, which is present on all doublets, i-SUB5-6 is only present on a single doublet, and o-SUB5-6 and the 1–2 bridge are even more restricted to sub-regions of this unique doublet. In addition, o-SUB5-6 and the 1–2 bridge form connections with higher periodicities and larger interfaces with the neighboring DMTs than N-DRC. Therefore, it is likely that the two SUB5-6 bridges and the 1–2 bridge contribute significant constraints to inter-doublet sliding in an asymmetric fashion. As a result, these connections might dramatically increase the stiffness of the connected DMT pair. Quasi-planar motility in 9+2 cilia and flagella is facilitated by restricting any off-axis bending, i.e., any bending not along the bending plane [20]. Increasing the stiffness of a DMT pair could efficiently resist lateral shear forces generated by the other DMT pairs and therefore define a bending plane perpendicular to the linked DMT pair. The different distributions of the sea urchin o-SUB5-6 and the *Chlamydomonas* 1–2 bridge might partly account for the asymmetric, cilia-like waveform of *Chlamydomonas* flagella (“breast stroke”), in contrast to the sinusoidal beating pattern of sea urchin flagella.

### The Unique DMT in axonemes Exhibits a Significantly Reduced Number of Dynein Arms

Axonemal dyneins are arranged in two rows on the DMTs, the ODAs and IDAs, and ATP hydrolysis-driven dynein motility powers sliding between adjacent DMTs [17], [18]. ODAs repeat every 24 nm along the length of the A-tubule of DMTs and contribute more than two-thirds of the sliding force [67]. The IDAs are more diverse, with seven distinct isoforms (structurally known as dyneins IA1-6 and IAX; biochemically known as dyneins a-g), and they are arranged along the A-tubule of DMTs with a 96-nm periodicity [43], [51], [54], [68]. Our cryo-ET analysis revealed clear structural differences on the unique DMT in the region in which axonemal dyneins are found on all other DMTs. These differences were conserved between *Strongylocentrotus* and *Chlamydomonas*. First, our doublet-specific averages revealed that the ODAs were replaced by o-SUB5-6 over almost the entire length of the *Strongylocentrotus* DMT5 and were either simply missing from *Chlamydomonas* DMT1 or replaced by the proximal 1–2 bridge. Despite exhibiting the same 24-nm periodicities, the observed 3D structure of sea urchin o-SUB5-6 shows no indications that they are modified ODAs (Figure 2; Movie S2). This feature is consistent with previous studies demonstrating that the o-SUB5-6 structures can not be decorated with antibodies against ODAs [69], [70]. Previous studies have also observed that ODAs are almost completely missing from *Chlamydomonas* DMT1 [15], [16], [71]. Second, several IDAs were also missing from the specialized DMT: dynein IA2-4 and IAX for *Strongylocentrotus*, and IA3, 4 and IAX for *Chlamydomonas.* In addition, the I1 dynein is also absent from the proximal quarter of *Chlamydomonas* DMT1, where the proximal 1–2 bridge is present. Thus far, none of the proteins forming i-SUB5-6, o-SUB5-6 or the proximal 1–2 bridge have been identified, and no evidence suggests that these inter-doublet linkers are constituted by the axonemal dyneins present in these regions on all other DMTs.

This significantly reduced number of dynein arms along the entire length of the specialized DMT of *Chlamydomonas* and sea urchin flagella should result in a dramatic reduction of the sliding force that could be produced between the unique DMT and its neighboring DMT. Therefore, the loss of dynein arms from the same particular DMT is consistent with the interpretation that the addition of inter-doublet links to the unique DMT prevents or limits sliding of *Strongylocentrotus* DMTs 5 and 6 or *Chlamydomonas* DMTs 1 and 2 against each other. This interpretation is also consistent with previous reports that DMT5 is permanently connected to DMT6 in mussel gill cilia [9], [11], [17]. Considering that these DMTs are located in a plane almost perpendicular to the bending plane, the limitation or prevention of sliding between these DMTs is expected to be critical for ciliary and flagellar quasi-planar waveforms. Thus, the unique features observed in *Strongylocentrotus* DMT5 and *Chlamydomonas* DMT1 are likely to be important for ciliary and flagellar quasi-planar motility. Previous studies also reported that in mammalian sperm, DMTs 5 and 6 do not exhibit inter-doublet sliding [72], [73]. These observations suggest that inter-doublet linkers similar to i-SUB5-6, o-SUB5-6 and/or the proximal 1–2 bridge are also present on DMT5 of motile mammalian cilia and flagella, indicating that the permanent linkage between these DMTs is a common feature of 9+2 cilia and flagella.

### Conserved Structures Suggest a Unifying Numbering Convention for DMTs in Eukaryotic axonemes

At present, two opposing numbering systems are used for designating the DMTs of 9+2 cilia and flagella. Historically, the numbering of DMTs was based on the relative position of each DMT with respect to the plane of the CPC [7], [8]) and/or the specific morphologies of mammalian outer dense fibers [74]. This convention was adopted for the cilia and flagella of many animals with a fixed (i.e., non-rotating) CPC [7], [9]–[13], [74], including sea urchin and mammalian spermatozoa. In this convention, the bending direction is perpendicular to the plane of the CPC, and runs through DMT1 and between the DMT pair DMT5-6 (Figure 1). In *Chlamydomonas* flagella, the CPC is not fixed at the flagellar base and rotates during flagellar beating [25], [75]. Therefore, another numbering system was proposed for *Chlamydomonas* based on the direction of the principal and reverse bends [15]. Because the DMT1 of mussel gill cilia (which have a typical 5–6 bridge) is located on the inside edge of the reverse bend, *Chlamydomonas* DMTs were indexed accordingly, such that DMT1 is also on the inside edge of the reverse bend [11], [15], [76]. Under these two DMT numbering systems, the unique DMTs in *Strongylocentrotus* and *Chlamydomonas* are indexed as DMT5 and DMT1, respectively. However, the conserved structural features identified in the present study strongly suggest that *Strongylocentrotus* DMT5 and *Chlamydomonas* DMT1 are corresponding, if not homologous, DMTs.

Considering that much of our knowledge about 9+2 cilia and flagella stems from studies of sea urchin sperm and *Chlamydomonas* flagella, it is important to correlate the axonemal organization, including the identity of the DMTs, between the two organisms. Historically, the second *Chlamydomonas* numbering system was based on the assumption that the directions of the principal/reversed bends are consistently related to corresponding DMTs. However, this assumption was challenged by a later study showing that sea urchin DMT1 is on the inside edge of the principal bend [12] and not of the reverse bend as in mussel gill cilia. Therefore, here, we unified the DMT numbering for 9+2 axonemes based on the conserved structural features (Figure 6), consistent with the original convention for most 9+2 axonemes, including sea urchin and mammalian flagella.

To avoid confusion between the two prevailing DMT numbering conventions, yet to encourage consistency with previous and future studies, we indexed the DMTs using the convention of the sea urchin numbering system, but with a “D” (for “doublet”) followed by Roman numerals instead of Arabic numbers. Under this DMT numbering system, the bending plane passes through DMT D-I and between DMTs D-V and D-VI. The unique DMT in *Strongylocentrotus* and *Chlamydomonas* is DMT D-V. By mapping previously described doublet-specific features onto this DMT numbering system, the doublet-specific RSJ of the third radial spoke would be absent from *Strongylocentrotus* DMTs D-I, D-II, D-V, and D-VI [13] and the beaks would be located in the B-tubules of *Chlamydomonas* DMTs D-I, D-V, and D-IX [15], [42]. Table 2 correlates the different DMT numbering systems and summarizes known and newly described doublet-specific features.

In conclusion, we have visualized the 3D structures of individual DMTs in the flagella of two evolutionarily distant organisms, *Chlamydomonas* and *Strongylocentrotus*. In both organisms, one of the nine DMTs exhibits unique structural features, which provide a structural basis for restricting the inter-doublet sliding between this unique DMT and the adjacent DMT, and can therefore define the bending plane in quasi-planar waveforms typical of 9+2 cilia and flagella. Revealing structural and functional differences among the nine doublets is an important step towards the long-term goal of understanding the inner workings of ciliary and flagellar motility.

## Supporting Information

Figure S1 **Comparison among all nine individual DMTs in the distal region of the** ***Strongylocentrotus*** **flagellum and** ***Chlamydomonas*** **axoneme.** After determining the doublet identities using doublet-specific axonemal markers (e.g., the 5–6 bridge in *Strongylocentrotus* or the B-tubule projection in *Chlamydomonas*), doublet-specific averages for DMTs 1–9 were generated by combining the axonemal repeats for each individual DMT from all tomograms of a particular strain. The structures of eight of the doublets, namely DMTs 1–4 and 6–9 in *Strongylocentrotus* and DMTs 2–9 in *Chlamydomonas*, look very similar at this resolution, whereas DMT5 in *Strongylocentrotus* and DMT1 in *Chlamydomonas* have unique structural features. The sea urchin DMT numbering is according to Afzelius [7] whereas the *Chlamydomonas* DMT numbering follows Hoops and Witman [15]. The tomographic slices are viewed from the front and show the same locations of the axonemal repeat as displayed in Figures 2E (for *Strongylocentrotus*) and 3E (for *Chlamydomonas*). Dynein IAX (dynein b/g) is absent from three DMTs in *Strongylocentrotus* and *Chlamydomonas*, namely DMTs 1, 5, and 9 (blue arrowheads); however, note that these DMTs are not homologous between the two species (see summary in Table 2). Scale bar (A): 25 nm.
(TIF)

Figure S2 **The ODAs connect to the adjacent DMT through narrow dynein stalks.** (**A–D**) Tomographic slices show the narrow connections of the small dynein coiled-coil stalks of ODAs in the sea urchin *Strongylocentrotus* (A and B) and the green alga *Chlamydomonas* (C and D) in cross-sectional (A and C) and longitudinal views from the bottom (B and D) (looking from the central pair towards the DMT). The red dashed lines in (A and C) indicate the locations of the tomographic slices shown in (B and D). The yellow arrowheads in (B and D) indicate the dynein stalks that connect to the adjacent doublet microtubule. Scale bar (A and B): 25 nm.
(TIF)

Figure S3 **Comparison of DMT1 between** ***Chlamydomonas*** **pWT and WT.** (**A–H**) Tomographic slices from the averaged repeats of pWT (A–D) and WT (E–H) axonemes show the i-SUB5-6 complex in cross-sectional (A, B, E, and F) and longitudinal views from the front (C, D, G, and H). The averaged pWT and WT structures are highly similar and show almost identical i-SUB5-6 and proximal 1–2 bridge structures. The red dashed lines in (A, B, E, and F) indicate the locations of the tomographic slices shown in (C, D, G, and H), respectively. Panels A–D correspond to Figure 4B, 3C, 4E, and 4F, respectively. The *Chlamydomonas* DMT numbers are according to Hoops and Witman [15]. Scale bars are 25 nm (scale bar in B valid for A, B, E and F; scale bar in D valid for C, D, G, and H).
(TIF)

Movie S1 **Comparison of the 3D structure of DMT5 to that of the other DMTs in the distal 7/8^th^ of the** ***Strongylocentrotus*** **flagellum (sea urchin).** An animated 3D visualization shows isosurface renderings of the averaged 96-nm axonemal repeats and reveals that the structure of DMT5 is distinctly different from that of the other doublets. Note that the SUB5-6 (sea urchin bridge connecting DMTs 5 and 6) structure consists of two parts: the inner i-SUB5-6 (orange) and the outer o-SUB5-6 complex (green).
(MPG)

Movie S2 **Comparison of the 3D structures of an ODA and the o-SUB5-6 complex in the flagellum of the sea urchin** ***Strongylocentrotus*** **.** An animated isosurface rendering shows that the 3D structure of o-SUB5-6 (green), which is only found on DMT5, is different from the ODA structure (purple) present on all other DMTs. At the beginning of the movie, the proximal end of the repeat is on the left side.
(MPG)

Movie S3 **Comparison of the 3D structure of DMT1 to that of the other DMTs in the distal three quarters of the** ***Chlamydomonas*** **flagellum.** An animated 3D visualization of isosurface renderings shows that the DMT1 structure is distinct from that of the other DMTs. The i-SUB5-6 structure (orange), which connects to the neighboring doublet, and the lack of certain dyneins (e.g., ODAs) are unique features of DMT1.
(MPG)
