# Supplementary figures and images for: One of the Nine Doublet Microtubules of Eukaryotic Flagella Exhibits Unique and Partially Conserved Structures
Source: PLoS One. 2012 Oct 10;7(10):e46494. doi: 10.1371/journal.pone.0046494 (PMC3468612; doi:10.1371/journal.pone.0046494)

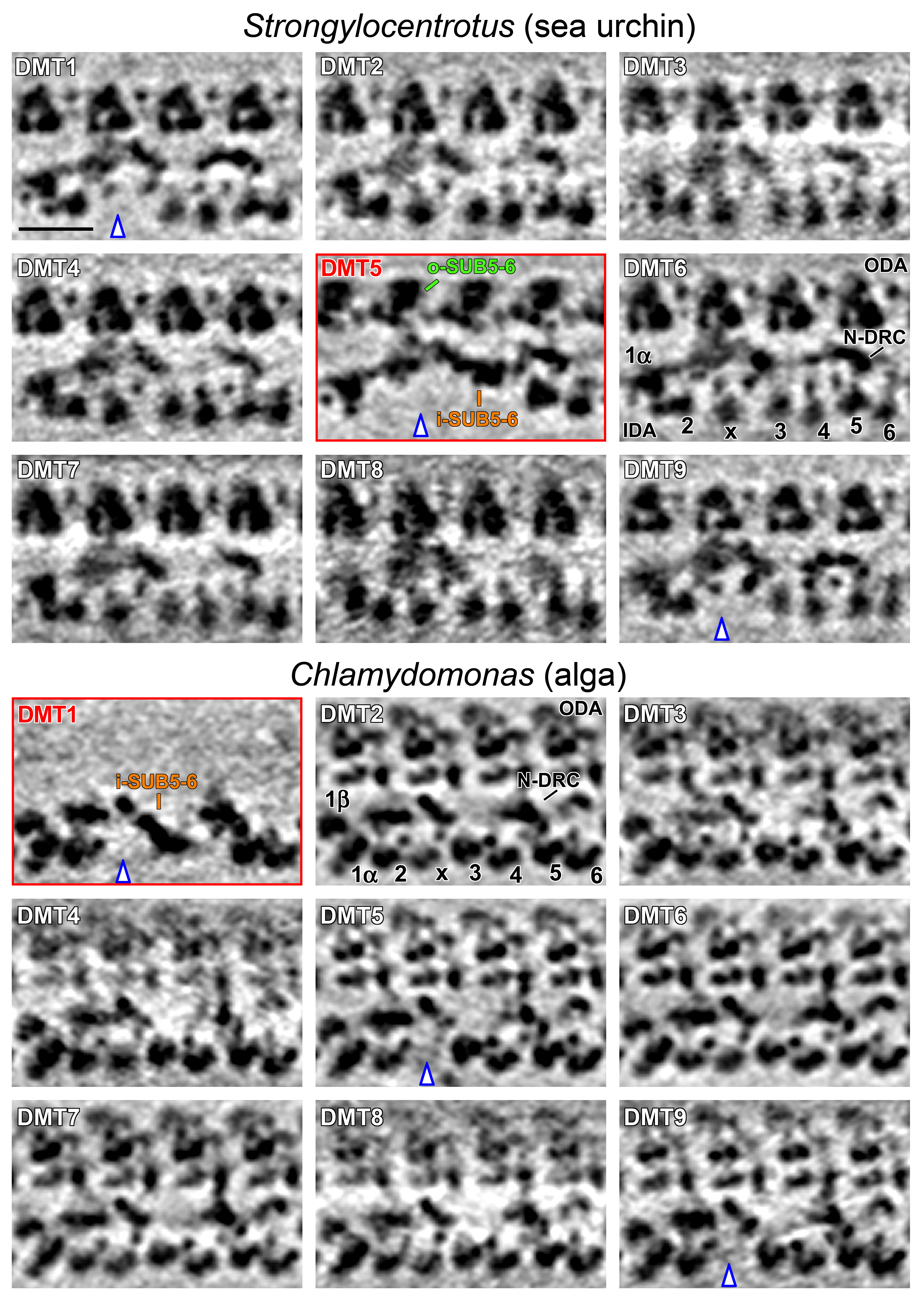

Supplement: Figure S1 — Comparison among all nine individual DMTs in the distal region of the Strongylocentrotus flagellum and Chlamydomonas axoneme. After determining the doublet identities using doublet-specific axonemal markers (e.g., the 5–6 bridge in Strongylocentrotus or the B-tubule projection in Chlamydomonas), doublet-specific averages for DMTs 1–9 were generated by combining the axonemal repeats for each individual DMT from all tomograms of a particular strain. The structures of eight of the doublets, namely DMTs 1–4 and 6–9 in Strongylocentrotus and DMTs 2–9 in Chlamydomonas, look very similar at this resolution, whereas DMT5 in Strongylocentrotus and DMT1 in Chlamydomonas have unique structural features. The sea urchin DMT numbering is according to Afzelius [7] whereas the Chlamydomonas DMT numbering follows Hoops and Witman [15]. The tomographic slices are viewed from the front and show the same locations of the axonemal repeat as displayed in Figures 2E (for Strongylocentrotus) and 3E (for Chlamydomonas). Dynein IAX (dynein b/g) is absent from three DMTs in Strongylocentrotus and Chlamydomonas, namely DMTs 1, 5, and 9 (blue arrowheads); however, note that these DMTs are not homologous between the two species (see summary in Table 2). Scale bar (A): 25 nm. (TIF) [file pone.0046494.s001.tif]

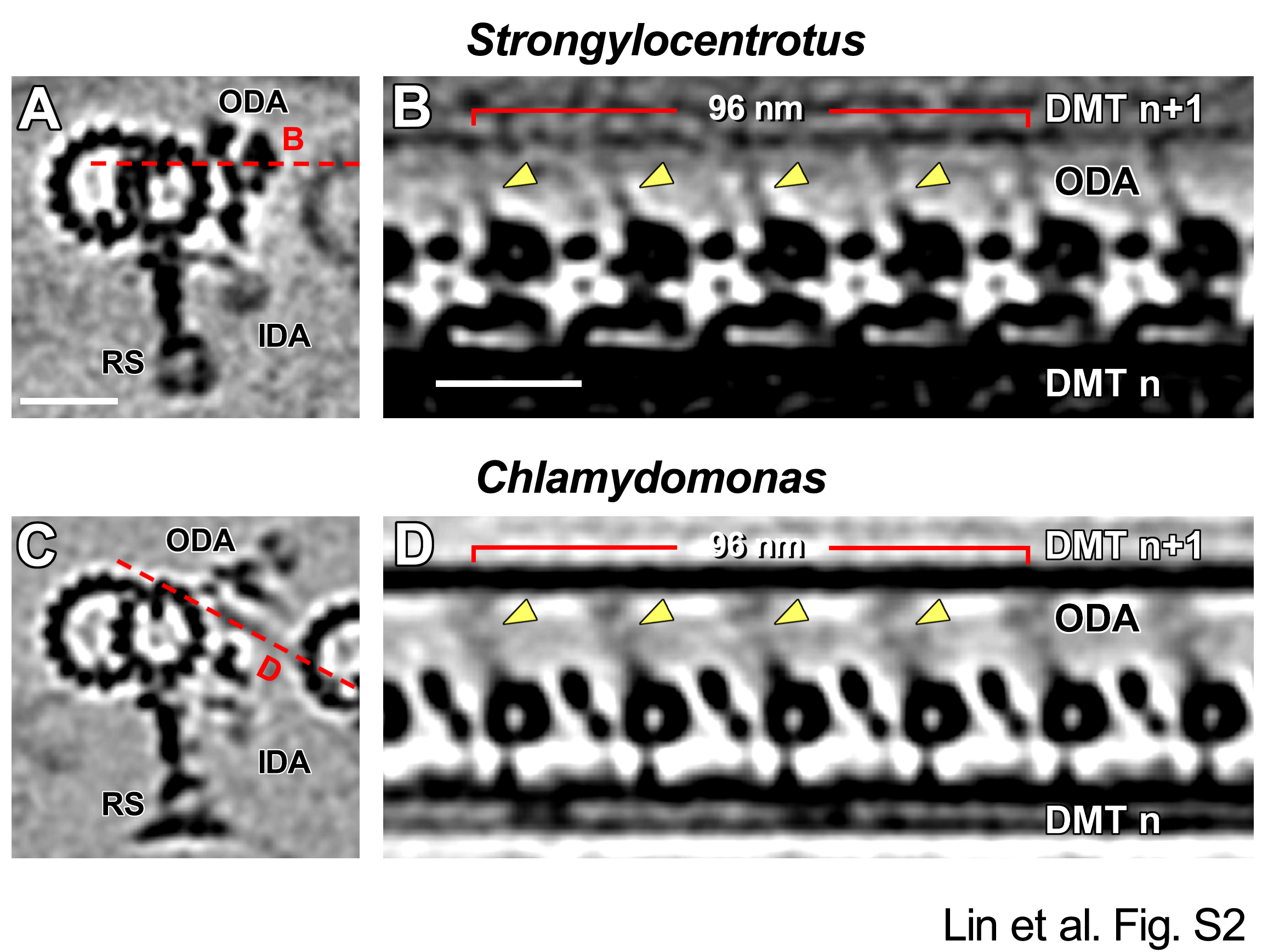

Supplement: Figure S2 — The ODAs connect to the adjacent DMT through narrow dynein stalks. (A–D) Tomographic slices show the narrow connections of the small dynein coiled-coil stalks of ODAs in the sea urchin Strongylocentrotus (A and B) and the green alga Chlamydomonas (C and D) in cross-sectional (A and C) and longitudinal views from the bottom (B and D) (looking from the central pair towards the DMT). The red dashed lines in (A and C) indicate the locations of the tomographic slices shown in (B and D). The yellow arrowheads in (B and D) indicate the dynein stalks that connect to the adjacent doublet microtubule. Scale bar (A and B): 25 nm. (TIF) [file pone.0046494.s002.tif]

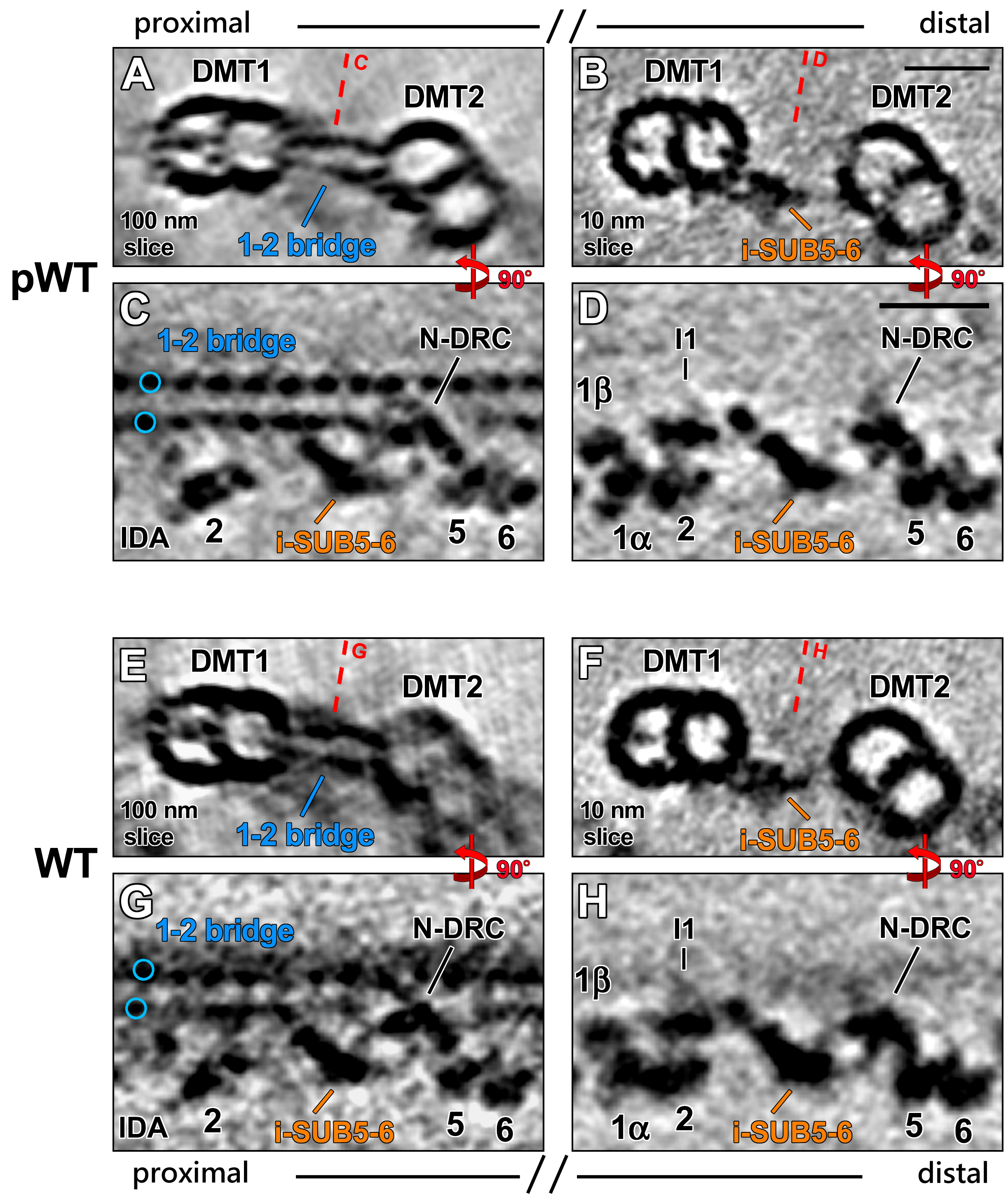

Supplement: Figure S3 — Comparison of DMT1 between Chlamydomonas pWT and WT. (A–H) Tomographic slices from the averaged repeats of pWT (A–D) and WT (E–H) axonemes show the i-SUB5-6 complex in cross-sectional (A, B, E, and F) and longitudinal views from the front (C, D, G, and H). The averaged pWT and WT structures are highly similar and show almost identical i-SUB5-6 and proximal 1–2 bridge structures. The red dashed lines in (A, B, E, and F) indicate the locations of the tomographic slices shown in (C, D, G, and H), respectively. Panels A–D correspond to Figure 4B, 3C, 4E, and 4F, respectively. The Chlamydomonas DMT numbers are according to Hoops and Witman [15]. Scale bars are 25 nm (scale bar in B valid for A, B, E and F; scale bar in D valid for C, D, G, and H). (TIF) [file pone.0046494.s003.tif]
